# Personality systems interactions theory: an integrative framework complementing the study of the motivational and volitional dynamics underlying adjustment to chronic pain

**DOI:** 10.3389/fpain.2024.1288758

**Published:** 2024-04-03

**Authors:** Anne Kästner, Frank Petzke

**Affiliations:** Department of Anesthesiology, Pain Clinic, University Hospital, Georg-August-University of Goettingen, Goettingen, Germany

**Keywords:** voluntary action control, self-regulation, pacing, avoidance and persistence behaviors, theoretical integration

## Abstract

In the endeavor to advance our understanding of interindividual differences in dealing with chronic pain, numerous motivational theories have been invoked in the past decade. As they focus on relevant, yet different aspects of the dynamic, multilevel processes involved in human voluntary action control, research findings seem fragmented and inconsistent. Here we present Personality Systems Interactions theory as an integrative meta-framework elucidating how different motivational and volitional processes work in concert under varying contextual conditions. PSI theory explains experience and behavior by the relative activation of four cognitive systems that take over different psychological functions during goal pursuit. In this way, it may complement existing content-related explanations of clinical phenomena by introducing a functional, third-person perspective on flexible goal management, pain acceptance and goal maintenance despite pain. In line with emerging evidence on the central role of emotion regulation in chronic pain, PSI theory delineates how the self-regulation of positive and negative affect impacts whether behavior is determined by rigid stimulus-response associations (i.e., habits) or by more abstract motives and values which afford more behavioral flexibility. Along with testable hypotheses, multimodal interventions expected to address intuitive emotion regulation as a central process mediating successful adaptation to chronic pain are discussed.

## 1 Introduction

People make remarkable efforts to adjust to a life with ongoing pain. While some individuals manage to maintain a high quality of life despite pain (i.e., “successful” adjustment), others feel impaired in their normal way of life, report high levels of suffering and dysfunction and develop psychopathological symptoms (e.g., depression). A multitude of biopsychosocial factors modulating the impact of persisting pain on psychophysical well-being and daily functioning have been described (1–3). Among these, pain-related activity patterns have received immense theoretical and empirical attention (4–7).

Cognitive-behavioral models originally developed for the psychotherapeutic treatment of anxiety disorders have been used and developed further to describe how acute pain evolves into chronic pain as a result of the avoidance of pain and pain-evoking movements (8–10). According to the Fear-avoidance Model (FAM) (11) of chronic pain, patients inflexibly adhering to the so-called fear-avoidance pattern tend to interpret pain as an indicator for structural tissue damage which induces excessive fear of pain, pain-evoking movements and reinjury. Moreover, they often anticipate a wide spectrum of negative consequences arising from the perceived physical disability such as impending unemployment and economic problems (a processing style denoted as “catastrophizing”). In the short term, avoidance behavior reduces fear which stabilizes this strategy by the operant mechanism of negative reinforcement (12). Especially in lack of an explanatory and functionally limiting structural tissue damage, avoiding movements or adopting unphysiological, restrictive postures and movement patterns may result in a variety of non-specific negative consequences in the long term: muscular imbalances, deteriorating muscle function, declining physical and mental resilience, reduced quality of life and psychological distress. Cognitive-behavioral treatment approaches addressing fear-avoidance behavior are often incorporated into interdisciplinary multimodal pain therapy (IMPT). They aim at cognitively restructuring unrealistic beliefs by bio-psycho-social knowledge transfer, cognitive techniques (e.g., socratic dialogue) and graded exposure to physiological movement patterns among other interventions (13).

Another subgroup of patients responds to pain in a way that is opposite to the evolutionary imperative of avoiding it. According to the Avoidance-Endurance Model (AEM) (5), these patients tend to suppress or minimize feelings of pain, pain-related thoughts and emotions and continue activities while ignoring signs of increasing physical and mental load (5, 6). Endurance behavior is thought to bear the short-term advantage of maintaining the usual level of functioning and still being able to achieve key performance goals. In the long run, the continuous self-overload may lead to an overuse and micro-damaging of musculature and joint structures, alienation from basic psychophysiological needs and, ultimately, psycho-vegetative exhaustion or depression. The therapeutic focus in this patient group lies on developing the ability to perceive and act on early warning signs of impending mental and or physical exhaustion to improve the balance between physical and mental load and regeneration.

Pacing behavior (e.g., reduction of work load or tempo; breaking tasks into smaller parts) is commonly regarded as an adaptive form of dealing with ongoing pain (7, 14). It can be considered quite established that the avoidance of pain or pain-provoking activities is intrinsically maladaptive in terms of pain chronification, daily functioning, and negative affect (4, 15–17). Mounting evidence indicates that pacing and endurance behavior, on the other hand, are multidimensional constructs that are neither adaptive nor maladaptive by nature (4, 15). Instead, preliminary research indicates that the type of goal underlying the respective behavior mediates its impact on functioning and well-being. If activity pacing serves the goal of retaining energy for valued activities, it seems to have a positive impact on daily functioning, mood and pain intensity (4, 18, 19). If activity pacing is used as a strategy to avoid pain, however, associations to poor outcomes seem to emerge that are comparable to those reported for activity avoidance (4). These initial findings already highlight the clinical relevance of understanding pain-related activity patterns from a motivational perspective, which emphasizes the function of a certain behavior with respect to the fulfillment of basic, needs (see also Section 2).

Interdisciplinary multimodal pain therapy (IMPT) is considered the treatment of choice when persistent pain has a negative impact on functioning and emotional well-being (20, 21). IMPT programmes are grounded in cognitive-behavioral principles and share the overarching goal of improving daily functioning [i.e., “functional restoration” (22)]. Although a standard cognitive-behavioral therapy (CBT) protocol for IMPT is unavailable to date (21, 23, 24), key interventions are exercise, education, cognitive restructuring, graded exposure, relaxation training and the teaching of different strategies of activity pacing such as the pain-independent interruption of tasks by short recovery breaks or a general reduction of work load or tempo (13, 25). IMPT has repeatedly been shown to be more effective than monomodal concepts, such as physiotherapy and pharmacological interventions (13, 26–28). A recent meta-analysis of longitudinal outcome evaluations of IMPT programmes found that 85% of the study cohorts included (*n* = 55) reported improvements from pre- to post-treatment across various outcome parameters. In 66% of the studies that included measurements at pre, post and follow-up time points, favourable pre-post effects could be maintained or even improved at follow-up. Thus, despite considerable advancement of diagnostic and therapeutic concepts over the past decades, research suggests that with our current, quite heterogenous concepts, about one third of the participants are not able to achieve and maintain clinically significant improvements from IMPT to follow-up (29). Goal conflicts (e.g., improving function vs. receiving social benefits) are therefore increasingly taken into account during the diagnostic process (30). Motivational aspects such as self-regulatory deficits, although highly relevant to personalization of intervention programs and clinical outcomes, are still mostly disregarded in routine clinical care, however (4).

While the prevailing cognitive-behavioral disorder models (e.g., AEM) have contributed substantially to unravelling mechanisms of pain chronification (31, 32), they are incomplete when it comes to explaining *why* individuals adopt certain behavioral strategies in dealing with pain in the first place. They are also insufficiently predicting *why* some individuals are able to change in response to interdisciplinary, multimodal treatment efforts while in others inflexible activity patterns persist (29). To enhance the effectiveness of intervention programs by driving their personalization, the descriptive assessment of pain-related activity patterns may be less important than understanding their function in the dynamic and complex process of goal generation, initiation, maintenance and coordination when facing pain. Consequently, a paradigm shift inspiring the integration of cognitive-behavioral disorder models with a motivational systems perspective could broaden the diagnostic and therapeutic approach for across a wide spectrum of pain disciplines (33–35). Ultimately, shedding light on the self-regulatory abilities that people need to cope with adversities in the process of change and goal-pursuit could promote the advancement and further personalization of interdisciplinary, multimodal treatment programs (36).

**Figure 1:**
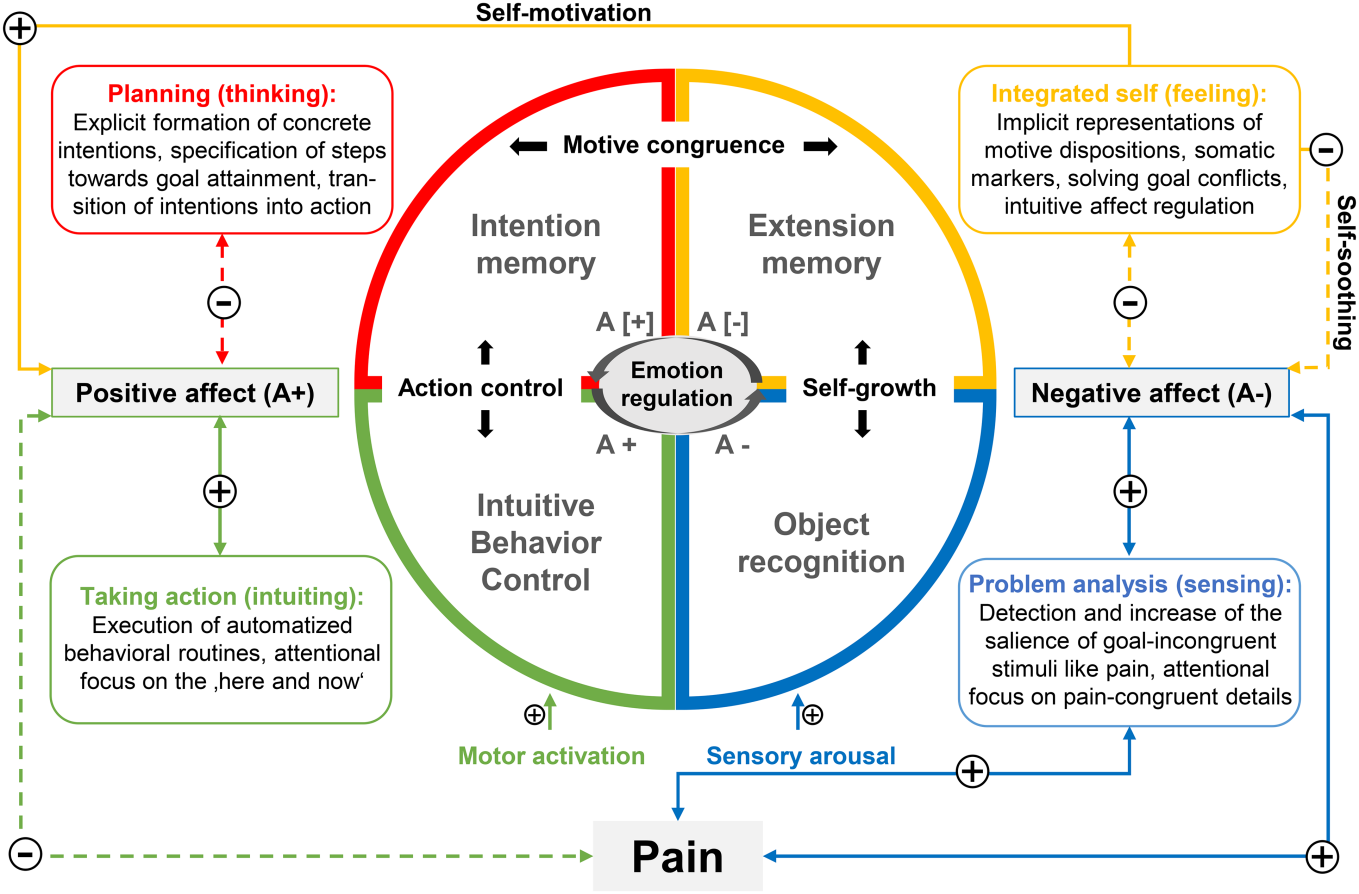
Illustration of the hypothetical modulatory influence of pain on the activation dynamics of the four cognitive systems proposed by PSI theory (adapted from Kuhl (38), p. 165 and Kazén and Quirin (37), p. 21). PSI theory conceptualizes two higher-order systems exerting dop-down influences on their respective elementary partner system. All systems can be characterized by a distinctive form of attentional and cognitive-affective processing referred to as functional profile. The cognitive systems are modulated by affect which places special emphasis on intuitive emotion regulation realized by extension memory. According to dual-process theories, positive and negative affect are mediated by two distinct neuropsychological systems (reward vs. punishment systems). Pain interfering with habitual ways of goal pursuit may be considered a stressful event placing high demands (e.g., obstacles, goal conflicts) on a person. Following PSI theory, demanding events are associated with reduced positive affect which is accompanied by an inhibition of intuitive behavior control, and an activation of intention memory. At the same time, pain can be conceived of as a threat to homeostasis which, as per PSI theory, is associated with increased negative affect, an activation of object recognition and an inhibition of extension memory. Activation thresholds of elementary systems are influenced by temperamental factors such as proneness to sensory arousal (introversion) or motor activation (impulsivity). Self-growth implies the integration of isolated painful experiences into extended experience networks (extension memory) which is fostered by the intuitive down-regulation of negative affect under threatening conditions (self-soothing). Action control implies the enactment of difficult intentions which is fostered by the intuitive up-regulation of positive affect under demanding conditions (self-motivation). An incongruence of implicit motive dispositions and explicit goal orientations (motive incongruence) results from the asymmetric activation of either intention or extension memory due to insufficient intuitive self-regulation of affect. Dashed lines and plus symbols indicate deactivating influences, solid lines and minus symbols indicate activating influences, square brackets indicate inhibition of positive (A+) or negative affect (A−).

In the endeavor to contribute to conceptual integration, this article suggests Personality Systems Interactions (PSI) theory as a scientifically precise and pragmatic overarching motivational framework (37–39) (Figure 1). By delineating its promising heuristic potential, we would like to stimulate the current discussion and the derivation of empirically testable hypotheses about the interplay of different motivational and volitional processes in the modulation of pain and its deleterious effects on daily functioning and quality of life.

The present article is organized into six sections. In the first section, we outline the defining premises of the motivational perspective on chronic pain. Next, we introduce PSI theory as a meta-framework explaining experience and behavior by the interactions of four motivational brain systems dynamically supporting important self-regulatory functions. We then discuss how PSI theory may inform our understanding of the motivational and volitional dynamics underlying adjustment to chronic pain by emphasizing the role of intuitive self-regulation of affect. The fifth section is dedicated to the clinical implications of the presented motivational systems perspective. We close by summarizing main aspects, discussing limitations and major challenges and by outlining potential future research directions.

## 2 Adjustment to chronic pain from a motivational perspective

From a motivational perspective, human beings constantly strive for the maintenance of dynamic equilibrium states. Homeostatic imbalances (i.e., deviation from individual set points) create physiological or psychological needs (40). Needs give rise to mental representations of goals designed to meet those needs (41). Various feedback loops act to initiate physiological and behavioral responses evolved to restore or maintain homeostasis by attaining need-fulfilling goals. Thus, homeostasis refers to an organism's ability to regulate a multitude of psychophysiological processes to keep internal states steady and balanced by motivating certain behavioral routines (40). Importantly, these regulatory processes mostly take place beyond conscious awareness.

Kazén and Quirin define “motivation” as the “extent to which our behavior is selected, directed, energized and maintained to fulfil basic human needs” (42), including basic psychological needs (e.g., acceptance, predictability, competence) (41) and motives (e.g., affiliation, achievement, power, autonomy) (41, 43). They refer to “volition” as central executive function coordinating different self-regulatory and self-control competencies relevant for the generation of need-fulfilling goals and intentions, their prioritization in a given situation and the shielding of a certain goal-directed behavioral routine from competing action tendencies (42).

Acute pain resulting from tissue damage is usually highly salient and has inherent aversive properties inducing negative affective states (44). From a motivational standpoint, pain interferes with habitual behavioral routines serving the attainment of valued goals. Consequently, ongoing pain introduces goal conflicts that have to be constantly resolved while going about daily routines (45). When people realize that previous need-fulfilling goals can no longer be achieved in the habitual way due to intense pain, physical impairments or reduced physical endurance, a complex process of adjustment is set in motion. This constant and fluid adaptation process may involve the disengagement from currently unrealistic goals and the definition of new self-compatible goals or rather the testing of new behavioral strategies of goal attainment (46–48).

## 3 The integrative potential of personality systems interactions (PSI) theory

In recent years, numerous motivational theories have been invoked to explain why people “choose” certain ways of dealing with chronic pain [for comprehensive overviews, see (34, 35)]. However, depending on the prevailing zeitgeist and research tradition, different models focus on specific aspects of the multilevel and dynamic process of human motivation. Thus, they offer quite different explanations for the same behavior, all of which most likely contain some truth (34, 35). Moreover, definitions and operationalization of constructs vary largely across experimental investigations rendering research findings rather fragmented and inconsistent (34, 35).

### 3.1 Seven sources of motivation

As a major contribution on its own, PSI theory provides a taxonomy that may contribute to organizing the fragmented research findings on the motivational underlyings of pain-related activity patterns. The taxonomy organizes the basic tenets of influential psychological theories of personality functioning by conceptualizing seven hierarchical levels of human motivation with increasing phylo- and ontogenetic complexity and behavioral flexibility (37, 49) (Table 1). The mechanisms outlined at each level are assumed to interact in real-time and in a context-sensitive manner. A comprehensive description of all seven levels with the respective empirical support is beyond the scope of the present article and is provided elsewhere (39). Table 1 represents a first attempt to allocate selected pain-related research findings to PSI theory's seven level taxonomy. How the different levels interact is further elucidated below when the four macrosystems are introduced.

**Table 1:** PSI theory's seven levels of motivation integrating major theories of human personality functioning.

Motivational levels, from complex to basic (assessment)	Within-level subsystems/processes	Related psychological/neuroscientific theories	References	Study population	Main research findings
Self-management [e.g., Volitional Components Inventory (50)]	Two modes of goal pursuit: *Self-control* (effortful, self-suppressive) *vs. self-regulation* (easy, self-maintaining, integrative)	*Self-determination theory* [SDT; Deci & Ryan (51)]	Kleinert et al. (52)	*N* = 173 non-patients, *N* = 81 patients with non-specific CBP	Types of autonomous and controlled motivation did not differ between patients and non-patients; controlled-convinced type of motivation was found to be most maladaptive with respect to body concept and adherence to exercise protocol
		*Dual-process model of coping* [Brandtstädter & Renner (53)]	Schmitz et al. (54)	*N* = 120 patients with chronic pain	A disposition to high accommodative flexibility (i.e., flexible goal adjustment) buffered negative effects of pain on psychological well-being
Cognitive processes [e.g., Word-Spelling Task and Remote Associates Task (55)]	*Analytical thinking* (conscious, step-by-step processing) *vs. feeling* (implicit, holistic, parallel processing)	*Dual-process theories of thought* [e.g., Evans et al. (40); Kahnemann (56)]	To our knowledge, studies on patients with chronic pain referring to *dual-process theories of thought* are not available to date.		
Motives [e.g., Operant Multimotive Test (57)]	Achievement, power, affiliation, autonomy, explicit goal orientations vs. implicit motives	*Consistency theory* [Grawe (58), motive incongruence]	Vincent et al. (59, 60)	*N* = 177 and *N* = 475 inpatients with chronic pain participating in IMPT	Higher need satisfaction (reduced motive incongruence) after IMPT mediated the association between the reduction in pain-related interference and psychological distress
Coping with stress [e.g., Action Control Scale (61)]	Behavioral control under stress mediated by the hippocampus, stress-dependent *regression (inhibition of top-down regulatory influences) vs. progression*	*Glucocorticoid-cascade-hypothesis* [Sapolsky et al. (62), *stress model of chronic pain* [Vachon-Presseau et al. (63)]	Review by Abdallah & Geha (64)	–	Chronic pain has been associated with hippocampal pathology [e.g., smaller hippocampal volume (63, 65)], data on dysregulation of HPA axis and cortisol level in chronic pain inconsistent (64)
		*Action Control Theory* [Kuhl (66)]	Buchmann et al. (67)	*N* = 536 patients with non-specific CBP	Distress-avoidance associated with high levels of dispositional state orientation (i.e., regressive form of coping with stress), eustress-endurance associated with high levels of dispositional action orientation (i.e., progressive coping)
Affect [e.g., PANAS (68), IPANAT (69)]	*Negative vs. positive affect* mediated by distinct neuropsychological systems (reward and punishment systems)	*Reinforcement sensitivity theory* [Gray (70)]	Review by Jensen et al. (71); Serrano-Ibañez et al., (72)	*N* = 516 patients with chronic Musculo-skeletal pain	Higher responsiveness of behavioral inhibition system (BIS) associated with fear-avoidance behavior, inconsistent findings regarding behavioral activation system (BAS) and persistence behavior; emotion regulation strategies mediate effects of BIS on experience (72)
Global arousal [e.g., Eysenck Personality Questionnaire (73)]	*Sensory arousal vs. motor activation* as global, non-stimulus related energetic basis for behavior (lowering of activation threshold of basic systems)	*Arousal theory of personality* [Eysenck (74)]	Review by Ramírez-Maestre et al. (75); Ramírez-Maestre et al. (76)	*N* = 96 patients with cancer pain	Fear-avoidance behavior is associated with neuroticism, introversion, anxiety sensitivity and experiential avoidance; high-level extraversion predicts use of active strategies of dealing with pain
Basic motor and perceptual systems (“habits”)	*Intuitive behavior control vs. object recognition*, rigid-stimulus-response associations	*Behaviorism* (e.g., Skinner)	Studies assessing pain-related activity patterns and catastrophizing		Rigid pain-related activity patterns can be considered habitual behaviors automatically triggered by environmental or interoceptive cues although they may not be contextually appropriate or in line with valued goals.

Examples of how the taxonomy may contribute to conceptually organizing research findings on the motivational underlyings of pain-related activity patterns are provided.

CBP, chronic back pain; IMPT, interdisciplinary multimodal pain therapy; IPANAT, implicit positive affect and negative affect test; PANAS, positive and negative affect schedule; PSI, personality systems interactions.

#### 3.1.1 Elementary levels of human motivation

At the lowest level affording the least behavioral flexibility, experience and behavior is determined by the basic processes of intuitive behavior control and object recognition that are automatically activated by environmental or interoceptive stimuli. The second level emphasizes individual differences in global arousal such as proneness to motor activation (i.e., impulsivity) and sensory arousal (i.e., neuroticism). The third level is consistent with the effect of behavioral approach and inhibition systems, BAS and BIS (70) on experience and behavior. Two-factor models such as Gray's reinforcement sensitivity theory (RST) have been frequently applied to pain-related activity patterns in recent years (70, 77). RST summarizes empirical evidence for the existence of two distinct neurophysiological systems [behavioral inhibition system (BIS) and behavioural approach system (BAS)] differentially modulating approach and avoidance behavior and associated cognitive-affective contents (71, 78). The predictions arising from the BIS-BAS model with regard to the motivational backgrounds of different pain-related activity patterns have a high degree of face validity, but have only been partially confirmed so far (72, 79, 80). A growing number of studies points towards an increased responsiveness of BIS in individuals who cognitively, emotionally, and behaviorally correspond to the FAM in dealing with chronic pain (80). The predicted associations between an activation of BAS and excessive persistence or adaptive forms of pain coping (i.e., pacing) have not yet been confirmed, however (71, 80, 81). This could be explained by the fact that complex organisms have gained degrees of freedom in action control and management in the course of evolution. They are not completely “at the mercy” of elementary brain systems rigidly responding to certain environmental or interoceptive stimuli. Voluntary action control in humans involves executive brain systems that can exert top-down regulatory influences on elementary motivational systems like BIS and BAS which provides humans with more behavioral flexibility (34, 82). In support of this, preliminary empirical evidence indicates that the emotion regulation strategy of cognitive reappraisal mediates the relationship between BIS and negative affect (72).

#### 3.1.2 Stress-dependent interface between elementary and complex mechanisms

The fourth level of the taxonomy (“coping with stress”) may be of particular relevance to pain research and treatment because pain can be conceived of as body-centered equivalent of stress (34). PSI theory conceptualizes this motivational level as a stress-dependent interface. It draws on neurobiological research on the pivotal role of the hippocampus in mediating whether elementary or complex cognitive processes take control of experience and behavior in a stress-level-dependent manner (62, 83). According to the glucocorticoid cascade hypothesis (62), under conditions of low to intermediate stress, behavior and experience is more likely to be modulated by higher-order (top-down) processes such as self-regulation, motives and goals (“progressive” form of action control). Under conditions of excessive stress, the hippocampus is inhibited beyond its critical glucocorticoid concentration. As a result, under excessive stress, the regulatory impact of higher-order brain functions (motives, goals and self-regulation) on action control is weakened. Consequently, behavior is increasingly determined by habits, basic temperament factors, or basic affective states (“regressive” form of action control)—even if these elementary processes do not match prior experiences, the self-concept, motive dispositions or goals. The individual sensitivity to stress is thought to vary substantially depending on prior exposure to stressful life events and genetic predispositions, amongst others (42). Conflicting findings regarding neuroanatomical and neurophysiological overlaps of chronic pain and stress may hint at a mediating role of interindividual differences in intuitively self-regulating stress levels (64).

#### 3.1.3 Complex levels of human motivation

The fifth level relates to the (relative) impact of the implicit social motives “affiliation, achievement, power, autonomy” on behavior. In PSI theory, the incongruence between implicit motives and goal orientations is referred to as motive incongruence which has been shown to play a role in the development of psychosomatic symptoms (84) (see section 4.3). At the sixth level, logical-symbolic, sequential processing (i.e., “analytical thinking”) is contrasted with associative thought based on parallel-processing networks (i.e., “holistic feeling”). To our knowledge, studies on patients with chronic pain referring to *dual-process theories of thought* (40) are not available to date. At the most complex level of his taxonomy, Kuhl distinguishes two coordinating volitional systems (“intention memory” and “extension memory”) which can exert top-down control of elementary processes (habits, temperament and affect). Two modes of goal pursuit can further be differentiated at this level: self-control and self-regulation (see also Table 1). At this level of complexity, a number of influential self-regulation theories have been used by Hasenbring and Kindermans (41) and others (35, 43) to conceptualize the motivational background of adaptive and maladaptive pain-related activity patterns. Against the background of self-discrepancy theory (SDT) (44), for example, pain behaviors are defined as “behavioral attempts to resolve discomfort and restore balance at the level of identity” (35). Among other convergences, SDT and PSI theory both recognize the duality of human self-regulation. SDT distinguishes autonomous from controlled self-regulation which is analogous to PSI theory's account of self-regulation and self-control [for an integrative review of SDT and PSI theory, see Koole et al. (85)]. Another motivational concept, the dual-process model of coping (45), contrasts the processing styles “assimilation” (i.e., tenacious goal pursuit) and “accommodation” (i.e., flexible goal adjustment) in response to the blocking of a certain goal. “Assimilation” is akin to Kuhl's definition of self-control while “accommodation” is akin to Kuhl's definition of self-regulation. Adapting the model to chronic pain, Van Damme and Kindermans (35) theorize that both avoidance and persistence behavior are expressions of the preferential use of the assimilative path of coping. They further argue that successful adaptation to a life with chronic pain, may involve the reassessment of goals or reappraisal of the situation, which would imply the reorganization (i.e., accommodation) of existing experience networks. A first empirical investigation indicates that a disposition to high accommodative flexibility (i.e., flexible goal adjustment) buffers negative effects of pain on psychological well-being. Predictions from the dual-process model need further empirical validation in chronic pain.

### 3.2 The functional profiles of four motivational macrosystems proposed by PSI theory

The mechanisms outlined at each of the seven levels of the taxonomy are assigned to four cognitive systems that are assumed to interact in real-time and in a context-sensitive fashion equipping humans with the potential of enormous behavioral flexibility. Rather than explaining interindividual differences in experience and behavior by analyzing cognitive contents (e.g., fear-avoidance and endurance beliefs), PSI theory takes a third-person perspective attributing experience and behavior to the relative activation and dynamic “communication” of two elementary (intuitive behavior control and object recognition) and two complex cognitive systems (intention and extension memory). These are conceptualized to take over different psychological functions pivotal for flexible and context-sensitive goal pursuit. Importantly, the interplay of complex systems and their respective elementary partner systems is facilitated by affective changes (Figure 1).

Anchored in the principles of affective neuroscience (46), PSI theory facilitates the integration and interpretation of biological and behavioral research on the shared mechanisms of chronic pain and emotional-motivational processing (47). It provides a functional analytics perspective on pain behaviors which, among other things, allows for:

• analyzing which internal regulatory processes take place when goal attainment is complicated by pain (i.e., analysis of motivational and volitional processes taking place during one goal episode in an individual)
• explaining why individuals oscillate between different pain-related activity patterns
• predicting which psychological functions are insufficiently developed in individuals who adhere to inflexible pain coping styles that have a negative impact on psychological well-being and everyday functioning
• deriving assumptions about how therapeutic interventions have to be designed to address certain brain systems supporting self-regulatory competencies
• In the following, we provide a brief overview of those functional aspects of the four macrosystems that may be most relevant to the understanding of the motivational and volitional dynamics of pain behaviors. We refrain from giving an exhaustive list of empirical findings in support of PSI theory's assumptions as these are provided elsewhere (37–39). For in-depth information on the neural mechanisms hypothesized to underlie the interactions between motivational levels and systems, see (39).

#### 3.2.1 Elementary (affective) brain systems

##### 3.2.1.1 Intuitive behavior control

This elementary system is responsible for the execution of behavioral routines that are genetically predisposed or have been automatized by learning (48). Intuitive behavior control can be conceived of as a basic form of intuition which subconsciously integrates contextual and interoceptive information relevant to orientation and movement (37). This function is hypothesized to rely on parallel-processing circuits centered on the dorsolateral striatum (86, 87). Positive affect (i.e., anticipated or obtained reward) facilitates the transition from strategic planning (intention memory) to action (88).

##### 3.2.1.2 Object recognition

When the pursuit of a need-fulfilling goal brings about more and more obstacles and setbacks, negative affect increases and/or positive affect decreases. Increased negative affect, in the case of goal failures, activates a system called “object recognition” which has evolved to detect “threats” to psychophysiological homeostasis. Relying on sequential-analytic bottom-up processing, it is responsible for the perception of goal-incongruent “objects” (39) such as pain exacerbations. The activation of object recognition is accompanied by an increase in negative affect and an attentional focus on pain-congruent information which may additionally exacerbate the inherent aversiveness and salience of pain (89). While object recogniton is activated, behavioral routines are interrupted (49). The temporary inhibition of intuitive behavioral control is adaptive whenever a retrospective, in-depth problem analysis is required. For the time of problem analysis, negative affect has to be endured. When the problem has been analyzed sufficiently, object recognition becomes deactivated. Then, a higher-order brain system with the potential of exerting top-down control on object recognition takes over (extension memory, see below).

#### 3.2.2 Complex (cognitive) brain systems

##### 3.2.2.1 Extension memory

Extension memory when activated in stressful situations leads to a decrease in negative affect (and pain aversiveness) by initiating a self-soothing response (90). Extension memory owes its name to extended associative neural networks that are capable of simultaneously processing a vast amount of information related to the self (e.g., needs, motives) (91) and the global context (e.g., needs of others, implicit social rules) (92, 93). The “results” of the parallel and implicit (i.e., mostly unconscious) computational processes executed by extension memory “enter” phenomenological consciousness as a kind of intelligent intuition. It provides human beings with a gut feeling of what is the right thing to do at a given moment (94). The so-called “self-system”, as a part of extension memory, receives and integrates interoceptive and proprioceptive input from the periphery. Body sensations help the self-system chose between different courses of action that have been tried before to satisfy a need [“somatic marker hypothesis” (95)]. Several lines of evidence converge on the notion that processes supported by extension memory [processing of emotions (96, 97) and body awareness] are lateralized to the right hemisphere (98, 99).

##### 3.2.2.2 Intention memory

The formation and maintenance of conscious intentions is supported by intention memory. PSI theory conceptualizes this higher-order executive system [allocated to the left frontoparietal cortex (100, 101)] as a “future-oriented” subsystem of working memory relying on explicit sequential-analytic processing (102). Intention memory is responsible for the implementation of intentions, the planning of action sequences, and the shielding of intentions from interfering action tendencies. Activation of intention memory is associated with the dampening of positive affect (103). Concurrently, pre-activated motor programs (intuitive behavior control) are inhibited to prevent premature execution until the necessary parameters of adaptive behavioral routines are specified and the appropriate opportunity for execution is encountered.

## 4 A PSI perspective on adjustment to chronic pain

Preliminary findings indicate that people “living well with pain” can be characterized by a high level of pain acceptance, a comparatively high quality of life and low pain-related psychological distress (104). They employ goal adjustment strategies such as flexible disengagement from currently inadequate goals and commitment to new goals (105). In a recent study relating different goal management strategies to pain-related activity patterns, flexible goal management, and commitment to new goals were associated with higher positive affect and persistence in finishing tasks despite pain (105).

According to PSI theory, an individual's capacity to intuitively (i.e., without deliberate efforts) regulate affective states in the face of emerging goal conflicts, obstacles and threatening life events (e.g., pain exacerbations) plays a pivotal role in the temporary or definitive disengagement from goals (106). Whether someone can “access” the brain system supporting the psychological function that best copes with a given situation depends on how well negative or positive affect can be endured and/ or intuitively down- or upregulated. From a PSI perspective, the maladaptivity of pain-related activity patterns may result from the biased activation of one or two cognitive systems due to insufficiently developed affect regulatory capacities which reduces behavioral flexibility and context sensitivity (39).

### 4.1 Flexible goal management and pain acceptance

A growing body of research relates chronic pain with various emotion regulation difficulties, including the up- and down-regulation of both positive and negative affect (107–111). Emotion regulation has even been suggested to serve as a transdiagnostic factor underlying chronic pain and co-morbidities such as opioid dependence, for example (107). Along the same lines, a mixed-methods study by Owens et al. (104) revealed that patients achieving well-being despite chronic pain possess the ability to deal with unpleasant feelings such as grief, shame and fear. They also manage to reevaluate their pain as coming with the potential of self-growth, enhanced relationships and an increased awareness of what is really important in life. Among other strategies, people living well with pain spontaneously use means of creative self-expression such as writing, painting, or making music to engage with their emotions and needs (104).

According to PSI theory, these individuals spontaneously employ strategies fostering “integrative emotion-regulation” (112) as a nondefensive form of down-regulating negative affect (i.e., self-soothing) which involves the broad experience networks of extension memory. A change from high to low negative affect (self-soothing) promotes the integration of isolated painful experiences into extension memory (37) (Figure 1). Exploring the personal meaning of a painful experience with respect to past experiences, values and motives can be conceived of as the functional basis of pain acceptance and self-growth (113). By providing intuitive answers to questions such as “What is really important to me?”, extension memory supports the resolution of goal conflicts and the definition of new self-compatible goals (114, 115). The processing style of intuitive judgement brings about global goals that are in line with multiple self-aspects and bear the advantage of intuitively and flexibly striving for different opportunities of goal attainment that emerge over time. In coping with pain exacerbations and stress, self-access can be assumed to facilitate the disengagement from goals that are not congruent with physical and emotional needs and the development of goals that satisfy “multiple constraints” (e.g., different needs, circumstances, past experiences). The process of pain acceptance can be assumed to be impaired when the intensity of stress or negative affect exceeds a critical threshold and access to extension memory (i.e., self-access) is inhibited (62, 83). When access to self-referential knowledge networks is hampered, individuals tend to mistake imposed duties or expectations of others for self-chosen goals, even though they do not match their own preferences- a phenomenon referred to as self-infiltration (116–118).

### 4.2 Goal maintenance despite pain

The enactment of intentions in the face of emerging difficulties such as recurring pain exacerbations (“i.e., action control”, Figure 1) requires the flexible interaction between intuitive behavior control and intention memory which is facilitated by positive affect. When it becomes apparent that a goal is more difficult to achieve than anticipated, positive affect is inhibited and frustration sets in. In this case, intention memory is activated to store the goal. By means of analytical thinking, intention memory generates solutions to the difficulties encountered and adjusts the plan of action to external or internal circumstances. When a solution has been found that increases the likelihood of goal achievement, a change from low to high positive affect (“self-motivation) affect removes the inhibition of intuitive behavior control and initiates action. Relying on substantial experimental evidence (119), it can be expected that an activation of intuitive behavior control while engaging in a task that is intrinsically rewarding attenuates pain salience and aversiveness. Consequently, especially in the face of a constant stressor such as chronic pain, it is essential to define goals that are supported by positive affect (“approach-oriented and self-compatible goals”) (90). In line with these predictions, studies drawing on value-expectancy models show that cues predicting reward reduce pain-related fear and avoidance behavior (120). According to PSI theory, goals that are in harmony with the implicit representations of needs, emotions, somatic markers and abilities (i.e., the ‘integrated “self” or “motive dispositions”) can be considered “self-compatible” or “motive congruent” (121).

### 4.3 Motive incongruence as a latent stressor in chronic pain

Achieving personal goals has been shown to be an important predictor of subjective well-being, but only if they are in line with motive dispositions (84). Since the pioneering work of McClelland, Koestner, and Weinberger (122), it can be considered well established that explicit goal orientations measured with questionnaires or goal surveys (e.g., Personal Project Analysis, PPA) have to be distinguished from implicit motives measured with operant motive tests, such as the Thematic Apperception Test or the Operant Multimotive Test (84). With reference to PSI theory, Baumann et al. (84) define motive dispositions as “implicit cognitive-emotional networks of possible actions that can be performed to satisfy basic social needs in a context-sensitive way across a variety of situations”.

According to PSI theory, implicit motives are represented in extension memory whereas explicit goal orientations are represented in intention memory. The ability to generate and implement motive-congruent goals depends on the quality of information exchange between extension and intention memory (Figure 1). Thus, in order to form goal orientations that match a broad spectrum of current physiological and psychological needs, intention memory has to “communicate” with extension memory. Both the asymmetric activation of intention memory (due to perseverating inhibited positive affect) and the inhibition of extension memory (due to perseverating negative affect) may impair the communication process leading to “motive incongruence”. People who constantly pursue goals that do not fit their own needs subject themselves to permanent stress without being aware of it. The pursuit of introjected goals is not facilitated by positive affect. It requires the effortful suppression of conflicting impulses to act which increases the risk for mental exhaustion (due to higher energy expenditure) and decreases the likelihood of behavior maintenance (39). Across all major motive domains (affiliation, achievement and power) using various methodological approaches, motive congruence has been reliably associated with increased life satisfaction, well-being and health (37). Converging evidence supports the idea that striving for “unwanted goals” (84) (“motive incongruence”) may act as a latent stressor which contributes to the formation of psychosomatic symptoms, among others (84).

## 5 Implications of PSI theory for IMPT

In IMPT, “functional restoration” or “improvement of physical and psychological load management” often serve as overarching therapy goals guiding the intervention process (28). Personalized therapeutic sub-goals such as “reduction of avoidance behaviors” or “improvement of spinal segmental stabilization” informed by the biopsychosocial perspective represent hypotheses on *what* patients have to change about their behavior to improve daily functioning, emotional well-being and quality of life, in the long run. For the duration of IMPT (usually three to six weeks), patients are taught certain strategies (stress regulation, relaxation techniques) and are instructed to increase their general activity level and follow certain physiotherapeutic exercises (28, 123, 124). After completion of therapy, they are faced with the challenge of integrating what they have learned into their daily routine in a self-directed manner. The formation of habits presumably increases the likelihood of behavior maintenance as they are supposed to compensate for transient volitional dips (125). Seminal work by Lally and colleagues (126) showed that habit formation is initially experienced as cognitively effortful until automaticity is reached. How long people need to automatize new health behaviors was found to vary between 18 and 254 days with one repetition per day and to depend on the consistency of practice as well as the complexity of the behavior (exercise vs. drinking) (126). The authors conclude that interventions designed to promote changes in unhealthy habitual behaviors should include continued support to help individuals perform a behaviour long enough for it to become automatized (126). Thus, concepts for long-term therapeutic support after inpatient or day patient IMPT to stabilize changes in attitudes and behavior are urgently needed (29). PSI theory may inform the development of concepts for IMPT after-care to maintain treatment effects by informing hypotheses on how affect-regulatory functions needed to reach difficult, long-term goals can be developed (34, 35, 67).

### 5.1 Toward a holistic understanding of “functional restoration”

Following the predictions emerging from PSI theory, for improving the long-term effectiveness of IMPT, it may be important taking into account individual deficits in the intuitive up-regulation of positive (self-motivation) and down-regulation of negative affect (self-soothing). Converging evidence points towards emotion regulation as a potentially unifying mechanism underlying diverse psychopathological symptom presentations, ranging from depression and anxiety disorders to chronic pain (107, 108, 110, 127). Recently, a multicenter RCT in patients with persistent medically unexplained physical symptoms revealed that therapy outcomes of CBT can be improved by emotion regulation training (128).

Many pain researchers and clinicians agree on the fact that impairments in intuitive self-regulation should inform case conceptualizations and treatment planning, including the hierarchization of therapy goals (34, 35, 67). Additionally, it may be clinically highly relevant to get an idea of how profound, persistent and generalized impairments in self-regulation are. Some individuals lose access to integrated self-representations only when they are severely stressed out. In others, however, self-access may be chronically impaired due to past, overwhelming relationship experiences that have not been well integrated into episodic memory and leave individuals in a state of constant hyperactivation (129).

### 5.2 Multimodal interventions that may foster intuitive self-access

Possibly the most important contribution of PSI theory in this context is that it allows the formulation of hypotheses on how interventions have to be designed to address the intuitive self-regulatory functions supported by the self-system (130). As can be derived from the functional profile of extension memory, self-access is mostly inaccessible to introspection and can thus not be influenced intentionally by using verbal, cognitive-behavioral strategies focussed on cognitive content. Consequently, to promote self-access and crosstalk between systems, multimodal approaches should be used that rely on the holistic-parallel, right-hemispheric processing of nonverbal, somatosensory-affective brain networks (131), such as experiential and relationship-based methods such as metaphors, visualization techniques, guided affective imagery, creative expression of emotions and body-oriented approaches which support a holistic perception of the body-mind connection (i.e., mind-body therapies). In addition to these concrete interventions, PSI theory suggests that therapeutic co-regulation is of extraordinary importance in fostering self-soothing abilities.

#### 5.2.1 Fostering self-soothing abilities by therapeutic co-regulation

According to attachment theory, during the first years of life, physiological and emotional needs of toddlers are directly translated into “self-expressive” behavior (e.g., crying) which is accompanied by an activation of the self-system. Sensitive and responsive primary caregivers recognize (“feel”) the needs underlying their child's expressive behavior and appropriately respond to those needs. PSI theory proposes that the ability to regulate one's emotions is acquired by internalization of the caregivers' regulatory behavior (e.g., calming down when child is upset) (38). The frequent and consistent co-activation of the self and subcognitive, affect-regulating systems is assumed to lead to a strengthening of the neuronal connections among those systems. As a result, activation of one of the systems later in life is sufficient to automatically activate the other (“system conditioning hypothesis”).

It can be derived from PSI theory's “system-conditioning hypothesis”, that the ability to intuitively down-regulate negative affect by co-activating the self-system and subcognitive, affect-regulating systems is promoted by the mirroring and validation of emotions by others rather than the teaching of emotion regulation strategies that rely on conscious deliberation (compare Gross' process model of emotion regulation) (132, 133). As IMPT is traditionally informed by cognitive-behavioral psychotherapy concepts, it mostly focusses on controlled emotion regulation such as enforced expression or cognitive reappraisal (66). These emotion regulation styles have been shown to be less beneficial with respect to volitional functioning, well-being, and high-quality relationships than integrative emotion regulation (IER) (112). Grounded in self-determination theory (SDT) (51), IER is based on adopting a mindful and accepting attitude towards one's emotional experience. Feeling one's emotions allows for an in-depth exploration of the experience's self-importance in terms of personal preferences, values and goals (112). Consistent with Kuhl's theorizing, SDT research indicates that autonomy-supportive elements of parenting such as empathizing with the child's feelings and helping to clarify experiences serve as a model for integrative emotion regulation and become internalized by the child (134).

Psychodynamic concepts emphasize implicit processes that operate outside of conscious awareness and cannot be influenced by deliberately applying certain techniques (112). In line with claims of leading experts in the field of emotion regulation (135), PSI theory offers an integrative perspective on emotion regulation as an “overarching meta-factor of therapeutic change” operating across psychotherapeutic approaches (135). While the conveyance of adaptive “controlled” emotion regulation strategies is slowly gaining importance in IMPT (110), the role of the therapeutic relationship in fostering intuitive self-regulation by activating the self-system continues to be underappreciated. In emotion-focused psychotherapy, for instance, the therapeutic relationship as a basis for the exploration of painful experiences is conceptualized as facilitating change processes by supporting integrative emotional processing (136). Consistent with Kuhl's “system conditioning hypothesis” and SDT, the “curative” effect of the therapeutic relationship is thought to arise from its affect-regulatory function that is internalized by the patient over time (136). A recent review summarizes the emerging evidence on the effectiveness of emotion-focused therapy in somatic symptom disorders and chronic pain (137).

We claim that, especially in the treatment of patients with severe deficits in intuitive self-regulatory abilities, all members of the multiprofessional team should be trained in offering a soothing, affect-tuned bond by being present, validating, accepting and authentic. This can be highly challenging in patients with a history of emotional neglect or childhood abuse who tend to transfer their negative relationship experiences to the therapeutic context. Attachment insecurity has been shown to be more prevalent in individuals with chronic pain as compared to the general population (138). Emerging evidence highlights associations of insecure attachment with poor adjustment to chronic pain (139–141) and poorer response to IMPT (142), although methodological issues (e.g., recall biases) complicate the interpretation of findings.

#### 5.2.2 Promoting adaptive interoceptive awareness

For people with chronic pain, aligning their goals with current physical needs may be crucial to avoid over- or underuse of muscle and joint structures. This places special emphasis on the ability to perceive, interpret and respond to internal body sensations. Thus, to be able to benefit from physiotherapeutic exercises in the long run, patients may need an intuitive sense for the right intensity and duration of a certain movement at the moment of practice. This requires “body awareness” which can be defined as a multi-dimensional construct referring to aspects of proprioception and interoception that enter conscious awareness (143). It has conceptual proximity to the construct of mindfulness (144) and has been shown to be compromised across a broad spectrum of chronic pain conditions (145–149).

Empirical evidence underscores the central importance of body awareness relayed via interoceptive pathways for the effective regulation of emotional responses (150). Interoception is defined as internal representation of all bodily sensations and lays the ground for emotional and motivational processing (151). Interoceptive inputs originating from various physiological systems are integrated into the limbic system, the anterior insula and the homeostatic sensorimotor cortex (150). Consistent with PSI theory's conceptualization of the self-system, Damasio and Cavalho theorize these brain structures to be involved in forming a meta-representation of the self which takes over the function of orchestrating finely-tuned regulatory responses (152). Bodily sensations (e.g., muscle tension) are among those signals assisting the self-system in choosing between different previously tried courses of action with varying degrees of “need satisfaction potential” (95). Consequently, interventions fostering body awareness should also facilitate access to intuitive self-regulatory functions supported by the self-system.

Enhancing body awareness has been proposed as mechanism of action by which mind-body approaches such as yoga, TaiChi, QiGong and mindfulness meditation positively impact psychosomatic symptoms and health in general (153). As a more therapeutic approach, body awareness therapies (BAT, e.g., Feldenkrais therapy) focus on promoting a non-judgemental and “mindful” way of perceiving the body as a whole and integrating it with other aspects of the self (including emotions) (154). This holistic approach has shown positive effects on pain experience, quality of movement and self-efficacy in psychiatric patients (155–158) as well as patients with specific and non-specific musculoskeletal pain including fibromyalgia (145–147, 149, 154, 159–161).

### 5.3 Improving interprofessional communication and coordination

In their recent meta-analysis on longitudinal outcome evaluations of IMPT in patients with chronic primary musculoskeletal pain, Elbers et al. observed that although most IMPT programmes shared the same theoretical foundation [i.e., the biopsychosocial model (162)], the interventions included were extremely heterogeneous, putatively unfolding their effects via different mechanisms of action. Also, the rationale for the application of certain therapeutic methods was not explicitly mentioned in most studies (29). Many experts in therapy research agree that the eclectic use of (psycho-) therapeutic techniques originating from different theoretical frameworks can be hindering to communication and progress of psychotherapy development (163, 164). Combining interventions of ACT and CBT, for instance, may be counterproductive as ACT assists people in opening up to unpleasant feelings, while CBT aims at gaining control and change pain-specific thoughts, feelings and sensations (165). The anticipation of potential synergies or dyssynergies when integrating multimodal interventions with varying degrees of theoretical and empirical validation may be even more challenging than integrating different psychotherapeutic interventions. Moving beyond learning-theory inspired behavior therapy techniques, PSI theory mechanistically integrates concepts and interventions from different psychotherapeutic traditions including psychodynamic approaches and body-oriented therapies. By conceptually linking disorder and behavior change theories, it may facilitate communication processes in multiprofessional teams, especially with respect to case conceptualizations and personalized therapy planning.

## 6 Discussion

This article presents Personality Systems Interactions (PSI) theory as an integrative motivational framework introducing a functional, third-person perspective on pain-related clinical phenomena. To date, there is a lack of integrative multilevel theories that bear the heuristic potential of deriving testable hypotheses about the interplay of multiple levels of motivation underlying adjustment to chronic pain. PSI theory which may fill this gap by integrating numerous motivational models and experimental findings from the fields of psychology, neurobiology and neuroscience [for a comprehensive overview, see (37, 39)].

In comparison to other multilevel theories that have been proposed in the context of pain, such as he Goal Centered, Self-regulatory, Automated, Social Systems Psychology (GRASSP) model proposed by Paul Karoly (33, 34), PSI theory reduces complexity for the sake of deriving testable hypotheses. PSI theory focuses on those self-control and self-regulatory functions most relevant to voluntary action control. The GRASSP model lists numerous attentional, affective and cognitive processes involved in human goal-directed behavior (166). Consistent with PSI theory, it views adaptation to chronic pain as an “emergent, time-bound, system-centered process” which is “continuously modulated by top-down and bottom-up self-regulatory mechanisms”. It is left unclear, however, how the different processes involved in self-control and self-regulation suggested by the GRASSP approach interact with each other in a given motivational context at the level of the individual. The authors themselves limit the applicability of their model by pointing out that the utility of self-regulatory processes suggested to play a role in voluntary action control “hinges upon their level of instrumental effectiveness”; when, why and how they are recruited” (34). Further, it cannot be derived from the GRASSP model how people form intentions and translate them into goals. Although the GRASSP model attempts to capture the complexity and dynamic nature of human motivation, to our opinion, its usefulness with respect to the definition of tangible research questions and the translation to clinical aims seems limited.

Based on PSI theory, we assume that individuals flexibly adjusting to a life with chronic pain have the ability to intuitively self-regulate positive and negative affect. Even under stressful circumstances, these individuals maintain self-access which enables them to define goals in accordance with a broad array of self-referential knowledge including body sensations (i.e., motive congruence). We further claim that pain-related activity patterns that are clearly maladaptive in nature (consistently associated with negative outcomes), such as pain avoidance or excessive persistence behavior (4), result from the biased and inflexible activation of one or two macrosystems involved in voluntary action control (Figure 1). Rigid and generalized pain avoidance behavior may arise from the chronic or stress-dependent regression to the functioning of elementary action-regulating systems such as object recognition (167).

Consistent with this, Pinto et al. propose a hypothetical model of fibromyalgia suggesting an overactive “threat” system and an underactive “soothing” system which may result in a continuous state of hyperactivation of the brain's “salience network” (129). Along the same lines, a recent investigation examined the influence of stress and dispositional self-regulation abilities [i.e., state vs. action orientation in coping with stress (66)] on pain-related avoidance and endurance response patterns (67) [as operationalized by the AEM (5)]. In line with our hypothesis, the authors found patients with distress-avoidance responses to be the most impaired with respect to the down-regulation of negative affect (i.e., high levels of state orientation). Eustress-endurance responders were characterized by low levels of stress and good intuitive self-regulation abilities (i.e., high levels of action orientation). Consistent with other work (4, 7, 105), the authors conclude that endurance responses may not be longitudinally maladaptive if they are associated with flexible goal adjustment and the pursuit of self-compatible (i.e., approach-oriented) goals facilitated by intuitive self-soothing and self-motivation abilities.

PSI theory posits that most of the mechanisms involved in the generation of goals and their ongoing flexible adjustment to the dynamically emerging pain-related functional disabilities are not accessible to consciousness. Consequently, the inability to change inflexible pain-related activity patterns may not be a matter of not *wanting* to change but rather a matter of not *being able* to. This may relieve patients with resistance to change from the stigma of failure and not being “motivated” or “lazy”. The presented theoretical framework complements prevailing cognitive-behavioral disorder models (e.g., AEM) describing dysfunctional attitudes and behaviors in dealing with ongoing pain by contributing knowledge about the abilities people need to maintain behavioral flexibility under challenging or stressful circumstances. The established efficacy of mind-body approaches in improving sleep and quality of life in patients with musculoskeletal pain may presumably be mediated by an improvement in volitional competencies which remains to be investigated.

### 6.1 Limitations

A limitation of the present article is its complexity and differentiation, especially for readers who have not yet dealt with motivation models in depths. Nevertheless, we are convinced that the clinical implications of the functional-analytics perspective offered by PSI theory can also be inspiring without having understood every model assumption in detail. Approaches of how to assess the psychological functions relevant to volitional action control that are of relevance for the diagnostic process an indication for IMPT are presented in the next section. Moreover, as the empirical validation of the presented hypotheses is still pending, assumptions are rather speculative in nature.

Another shortcoming concerns that we did not explicitly elaborate on the interaction of PSI theory with social barriers to initiate change, such as low socioeconomic status, racism and practical barriers (168). It can be assumed that these constitute additional stressors that further increase demands on self-regulatory functions and the ability to integrate conflicting self-relevant experiences in order to develop and maintain a coherent sense of self.

### 6.2 Future research directions and major challenges

The basic tenets of PSI theory have been validated by an extensive body of experimental and neurobiological research (38, 39). The predictions emerging from PSI theory regarding the functional architecture of behaviors that promote or complicate adapting to a life with pain remain to be empirically tested, however. The individualized assessment of functional mechanisms resulting from the dynamic interaction of the four personality systems proposed by PSI theory is made possible by innovative and well-validated measurement methods. The “Evolvement-Oriented System Diagnosis” (EOS) allows for measuring the different levels of psychological functioning relevant to motivation and volition including implicit and explicit motives and affect and self-management competencies (49, 169). Some examples of studies using self-report questionnaires and experimental paradigms operationalizing the main constructs at each level are listed in Table 1.

Longitudinal observational studies or experimental designs are needed to examine whether pain leads to an altered perception of the self and associated processes (e.g., self-regulation) or whether impaired intuitive self-regulation and impaired self-access under stress contribute to the development of chronic pain. The role of chronic latent stressors such as motive incongruence or need frustration in mediating the relationship between poor self-regulation under stress and adjustment to chronic pain should be further explored in future studies. Research into the relative impact of adverse childhood experiences, attachment styles and temperamental factors (e.g., proneness to BIS and/or BAS activation) on self-regulatory functions would also contribute to advancing the personalization of diagnostics and therapeutic approaches.

Once sufficient empirical evidence corroborates the central role of the mechanisms proposed by PSI theory in chronic pain, the assessment of the relevant constructs should be integrated into the diagnostic process. In the assessment phase usually preceding IMPT, individual constellations of biopsychosocial risk factors are determined by clinical interviews and self-report questionnaires that guide the definition of therapy goals (13). The assessment of psychological functions important to flexibly adapt behaviors to fluid internal and external circumstances is of major relevance to change processes. The valid and reliable assessment of volitional and motivational competencies is a major challenge not only in terms of limited time resources, however. Various self-report questionnaires assessing similar self-regulatory constructs have emerged from different theoretical backgrounds in the last years (170, 171). The DSM-5 Alternative Model for the Assessment of Personality Disorders (AMPD) comprises a dimensional rating of basic psychological functions including self-regulation (172). The respective self-report instrument, the DSM–5 Levels of Personality Functioning Scale, has good psychometric qualities (171). Interestingly, the Operationalized Psychodynamic Diagnosis System (OPD) defines similar psychological capacities necessary for adaptive personality functioning, such as self-awareness and affect regulation (173). A 12-item short version (OPD-SQS) has recently been published (170). It contains items addressing self-regulatory abilities which have been shown to partially mediate the relationship between adverse childhood experiences and somatic symptom load and psychological distress (174). It would be interesting to assess convergent and discriminant aspects of construct validity of the volitional components inventory (50) which emerged from PSI theory and the mentioned self-report questionnaires addressing similar constructs. On the long run, new self-report questionnaires should be developed for patients suffering from musculoskeletal pain assessing those volitional and motivational functions most relevant to this patients group.

A major methodological challenge concerns the valid assessment of compromised self-access (130). PSI theory defines “the self” as an extensive network operating according to connectionist, parallel-processing principles (93) rendering it largely inaccessible to conscious deliberation (175). Consequently, people who are alienated from the self are not aware of the true extent of their alienation in most cases (130). Consequently, profound deficits in intuitive self-regulation may not become clinically apparent at first sight. In a similar vein, introspective reports likely tap into consciously represented aspects of the self-concept rather than the broad experience networks underlying implicit self-knowledge (176). New questionnaires for the assessment of motivational and volitional constructs in chronic pain should be validated using behavioral or psychophysiological measures of implicit emotion regulation [e.g., reaction times in affective priming tasks (177) or heart rate variability (178)]. Informed by PSI theory, several non-reactive measures of self-access have been developed over the last decades. Baumann et al. (130) provide a comprehensive overview of different measures of self-access that address different aspects (consistency-based measures) and processing characteristics (latency-based measures) of the self-system. To our knowledge, these measures and paradigms have not yet been applied to patients with chronic pain, which opens up an intriguing new field of research.

## Data Availability

The original contributions presented in the study are included in the article/Supplementary Material, further inquiries can be directed to the corresponding author.
